# Extracellular Vesicle-Mediated IL-1 Signaling in Response to Doxorubicin Activates PD-L1 Expression in Osteosarcoma Models

**DOI:** 10.3390/cells11061042

**Published:** 2022-03-18

**Authors:** Su Yati, Atiruj Silathapanasakul, Chakrarin Thakaeng, Mayuree Chanasakulniyom, Napat Songtawee, Sureerut Porntadavity, Peraphan Pothacharoen, Dumnoensun Pruksakorn, Prachya Kongtawelert, Pa-thai Yenchitsomanus, Theerawut Chanmee

**Affiliations:** 1 Department of Clinical Chemistry, Faculty of Medical Technology, Mahidol University, Phutthamonthon, Nakhon Pathom 73170, Thailand; su.yat@student.mahidol.ac.th (S.Y.); atiruj.sit@student.mahidol.edu (A.S.); chakrarin.tha@gmail.com (C.T.); mayuree.chn@mahidol.ac.th (M.C.); napat.son@mahidol.ac.th (N.S.); sureerut.por@mahidol.ac.th (S.P.); 2 Thailand Excellence Center for Tissue Engineering and Stem Cells, Department of Biochemistry, Faculty of Medicine, Chiang Mai University, Chiang Mai 50200, Thailand; peraphan.p@cmu.ac.th (P.P.); prachya.kongtawelert@gmail.com (P.K.); 3 Musculoskeletal Science and Translational Research Center (MSTR), Faculty of Medicine, Chiang Mai University, Chiang Mai 50200, Thailand; dumnoensun.p@cmu.ac.th; 4 Division of Molecular Medicine, Research Department, Faculty of Medicine Siriraj Hospital, Mahidol University, Bangkok 10700, Thailand; ptyench@gmail.com

**Keywords:** chemoresistance, extracellular vesicles, immunosuppression, interleukin 1, osteosarcoma, programmed cell death ligand 1

## Abstract

The expression of programmed cell death ligand 1 (PD-L1) in tumors is associated with tumor cell escape from T-cell cytotoxicity, and is considered a crucial effector in chemoresistance and tumor relapse. Although PD-L1 induction has been observed in patients after chemotherapy treatment, the mechanism by which the drug activates PD-L1 expression remains elusive. Here, we identified the extracellular vesicles (EVs) as a molecular mediator that determines the effect of doxorubicin on PD-L1 expression in osteosarcoma models. Mechanistically, doxorubicin dependently stimulates the release of extracellular vesicles, which mediate autocrine/paracrine signals in osteosarcoma cells. The recipient cells were stimulated by these EVs and acquired the ability to promote the expression of inflammatory cytokines interleukin (IL)-1β and IL-6. In response to doxorubicin, IL-1β, but not IL-6, allowed- osteosarcoma cells to promote the expression of PD-L1, and the elimination of IL-1β/IL-1 receptor signaling with IL-1 receptor antagonist reduced PD-L1 expression. Together, these findings provided insights into the role of EV release in response to chemotherapy that mediates PD-L1 expression via the IL-1 signaling pathway, and suggested that the combination of a drug targeting IL-1 or PD-L1 with chemotherapy could be an effective treatment option for osteosarcoma patients.

## 1. Introduction

Cell-to-cell communication is a complex mechanism that coordinates cellular events among different cell types under physiological and pathological conditions. In the context of cancer, this process is mediated by direct cell contact, the secretion of soluble factors, and the release of extracellular vesicles (EVs) [1,2]. EVs are broadly classified into three major subtypes according to their origin and size: exosomes (30–120 nm), microvesicles (0.1–1.0 µm), and apoptotic bodies (0.8–5.0 µm) [1]. Cancer-derived EVs modulate various biological events by transferring their contents into recipient cells or by ligand/receptor interaction, thereby contributing to multiple aspects of tumor progression such as invasion, angiogenesis, metastasis, and immune evasion [3,4]. Cancer cells utilize distinct pathways to regulate EV secretion via activated or overexpressed EV regulator proteins, including small GTPase, syntenin, heparinase, and SNARE [5]. Recent studies have proposed that chemotherapeutic agents strongly stimulate the release of EVs, enhancing chemoresistance and the prometastatic capacity [6,7,8,9], although the biological process of chemotherapy-induced EV release remains to be determined.

Inflammation promotes tumor progression, and supports metastatic spread and chemoresistance [10,11]. The contribution of inflammatory cytokines in mediating tumor recurrence has been extensively studied [12,13]. The expression of IL-6, IL-8, and IL-1β is strongly associated with tumor relapse, and protects the cancer cell from chemotherapy by enhancing multidrug resistance protein 1 and antiapoptotic protein expression [14,15,16,17]. Currently, chemotherapy exposure markedly increases the release of inflammatory cytokines such as IL-6 and IL-8 in the tumor microenvironment, promoting cancer cell survival and self-renewal signaling of cancer stem cells, which are crucial for drug resistance and relapse [18,19]. Additionally, increased cancer programmed cell death ligand 1 (PD-L1) expression following chemotherapy has attracted more attention [20,21,22] because PD-L1 signaling is a critical immune checkpoint in antitumor immunity and chemoresistance. Given these important roles and consequences, PD-L1 is regulated at the transcriptional and translational levels by cytokines such as interferon gamma (IFN-γ), IL-4, IL-6, tumor necrosis factor alpha (TNF-α), IL-1β, IL-1α, and transforming growth factor beta (TGF-β) [23,24,25]. In addition to histological examination, the upregulation of PD-L1 was observed following chemotherapy in various tumor tissues, particularly osteosarcoma [26], non-small-cell lung cancer [22], bladder cancer [21], and breast cancer tissues [20]. Consequently, high PD-L1 levels are considered to be correlated with a poor prognosis and tumor recurrence [27,28], suggesting that upregulated PD-L1 may protect surviving cancer cells from the antitumor immune response and chemotherapy-induced cell death. However, whether and how chemotherapeutic agents regulate PD-L1, which is essential to cancer cell survival under critical conditions, remain unclear.

Here, we demonstrate that chemotherapeutic agents such as doxorubicin induce EV release, stimulating neighboring cancer cells to express IL-6 and IL-1β in osteosarcoma. We show that a significant increase in PD-L1 following doxorubicin treatment is achieved via the secretion of IL-1β, the inhibition of which abrogates PD-L1 expression. IL-1β may be required for cancer recurrence by maintaining PD-L1 expression during chemotherapy. These findings suggest that the induction of inflammatory cytokines and PD-L1 may represent potential therapeutic targets for osteosarcoma in combination with chemotherapeutic agents in preventing tumor relapse.

## 2. Materials and Methods

### 2.1. Cell Lines and Culture

The human osteosarcoma cancer cell lines MG63 and 143B (ATCC) were maintained in Dulbecco’s Modified Eagle Medium (DMEM; Gibco, Grand Island, NY, USA) supplemented with 10% heat-inactivated fetal bovine serum (FBS; Gibco, Grand Island, NY, USA) and 1% penicillin–streptomycin (Gibco, Grand Island, NY, USA) at 37 °C with 5% CO_2_ in a humidified incubator.

### 2.2. Cell Viability Assay

Osteosarcoma cells (3 × 10^3^ cells) were seeded in 96-well tissue culture plates and treated with various concentrations of doxorubicin (Abcam, Cambridge, UK) for 4 days, and then were subjected to cytotoxicity testing with an MTS assay (Promega, Madison, WI, USA) according to the manufacturer’s instructions.

### 2.3. Preparation of 143B/DOX and MG63/DOX Cells

The 143B and MG63 cells (2 × 10^5^ cells) were treated with 40 nM doxorubicin for 4 days, washed twice with PBS, and continually cultured in doxorubicin-free media for 10 days; these cells were named 143B/DOX and MG63/DOX, respectively, and were used in subsequent experiments.

### 2.4. Preparation of Conditioned Medium (CM-DOX)

Osteosarcoma cells (1 × 10^6^ cells) were treated with 40 nM doxorubicin for 4 days, washed with PBS twice, and then incubated with doxorubicin-free media for 3 days. The culture media were collected, centrifuged, and stored at −80 °C.

### 2.5. Isolation of EVs

EVs were isolated from the cell culture supernatants of osteosarcoma cells treated with doxorubicin. Briefly, osteosarcoma cells (1 × 10^6^ cells) were treated with 40 nM doxorubicin and cultured for 1, 2, and 4 days. The culture media were centrifuged at 300× *g* for 10 min to remove cell debris, and then the supernatants were collected and centrifuged at 20,000× *g* and 4 °C for 30 min to pellet the EVs. The EV pellets were then washed twice with PBS at 20,000× *g* and 4 °C for 30 min. After the final centrifugation, the isolated EVs were resuspended in filtered PBS, and the protein concentration was measured using the Pierce^TM^ BCA Protein Assay Kit (Thermo Scientific, Rockford, IL, USA).

### 2.6. Treatment of Osteosarcoma Cells with Recombinant Proinflammatory Cytokines

To determine the effects of IL-1β and IL-6 on PD-L1 expression, human osteosarcoma cells were seeded in 6-well plates at a density of 1 × 10^5^ cells/well. Next, the cells were treated with 10 ng/mL of recombinant human IL-1β (rIL-1β; PeproTech, Cranbury, NJ, USA) or 20 ng/mL of recombinant IL-6 (rIL-6; StemCell^TM^ Technologies, Vancouver, BC, Canada). After 24 h, the cell lysates were collected for qRT-PCR. For flow cytometry analysis, the osteosarcoma cells were treated with recombinant human IL-1β or IL-6 for 48 h and analyzed for cell surface PD-L1 expression.

### 2.7. Treatment of Osteosarcoma Cells with CM-DOX and EVs

Osteosarcoma cells (1 × 10^5^ cells) were seeded in 6-well plates and treated with CM-DOX in the presence or absence of 100 ng/mL of IL-1 receptor antagonist (IL-1RA; BioLegend, San Diego, CA, USA) for 24 h and 48 h to determine PD-L1 gene and protein expression using qRT-PCR and flow cytometry analysis, respectively. For EV treatment, osteosarcoma cells (1 × 10^5^ cells) were treated with 100 µg/mL of EVs for 24 h in the presence or absence of 100 ng/mL of IL-1RA, and PD-L1 gene expression was determined by qRT-PCR.

### 2.8. RNA Isolation and Quantitative Real-Time PCR (qRT-PCR) Analysis

Total RNA was isolated from the cells using the Geneaid Total RNA Mini Kit (Geneaid, New Taipei, Taiwan) according to the manufacturer’s instructions. RNA was quantified using a NanoDrop 2000 spectrophotometer (Thermo Fisher Scientific, Waltham, MA, USA), and 500 ng of total RNA was subjected to cDNA synthesis using the iScript Reverse Transcription Supermix (Bio-Rad, Hercules, CA, USA). The qRT-PCR was performed using Luna^®^ Universal qPCR Master Mix (New England BioLabs, Ipswich, MA, USA) and analyzed using the CFX96 Real-Time Thermocycler detection system (Bio-Rad, Hercules, CA, USA). The relative mRNA expression of each target gene was normalized to GAPDH expression. The following PCR primers were used in this study: GAPDH: forward, 5′-AGCCACATCGCTCAGACAC-3′ and reverse, 5′-GCCCAATACGACCAAATCC-3′; IL-1β: forward, 5′-GGACAAGCTGAGGAAGATGC-3′ and reverse, 5′-TCGTTATCCCATGTGTCGAA-3′; IL-6: forward, 5′-CCTGAACCTTCCAAAGATGGC-3′ and reverse, 5′-TTCACCAGGCAAGTCTCCTCA-3′; IFN-γ: forward, 5′-GAGTGTGGAGACCATCAAGGA-3′ and reverse, 5′-TGTATTGCTTTGCGTTGGAC-3′; TNF-α: forward, 5′-GCCCATGTTGTAGCAAACCC-3′ and reverse, 5′-TATCTCTCAGCTCCACGCCA-3′; TGF-β: forward, 5′-TTGCTTCAGCTCCACAGAGA-3′ and reverse, 5′-TGGTTGTAGAGGCAAGGAC-3′; and PD-L1: forward, 5′-GGTGCCGACTACAAGCGAAT-3′ and reverse, 5′-AGCCCTCAGCCTGACATGTC-3′.

### 2.9. Flow Cytometry

Osteosarcoma cells (3 × 10^5^ cells) were harvested and stained with antimouse PD-L1 (CD 274) antibodies (1:400 dilution; BioLegend, San Diego, CA, USA) for 1 h on ice, and then washed and incubated with Alexa Fluor 488 antimouse antibody (1:1000 dilution; BioLegend, San Diego, CA, USA) for 1 h. The cells were washed and then resuspended in cold PBS supplemented with 1% FBS. In total, 30,000 cells were analyzed using a BD FACS Canto II cytometer (BD Biosciences, Oxford, UK). The data were analyzed using FlowJo v10.7.1 software (TreeStar Inc., Ashland, OR, USA).

### 2.10. Enzyme-Linked Immunosorbent Assay (ELISA)

Cell culture supernatants derived from osteosarcoma cells treated with 40 nM doxorubicin or 50 µg/mL of EVs were collected. Next, the protein level of the proinflammatory cytokines IL-1β and IL-6 were measured quantitatively with a sandwich enzyme-linked immunosorbent assay using the Human IL-6 ELISA MAX^TM^ Deluxe Kit (BioLegend, San Diego, CA, USA) and Human IL-1β kit (Invitrogen, Vienna, Austria) according to the manufacturers’ instructions.

### 2.11. Quantification and Characterization of Extracellular Vesicles

The BD FACS Canto II cytometer was calibrated to detect EVs by comparing EVs with different fluorescent bead sizes (0.22 µm and 1.33 µm) (Spherotech, Lake Forest, IL, USA). The beads were mixed with EVs and analyzed with a flow cytometer to generate a gate for the following experiment.

EVs was quantified by BD Trucount^TM^ Fluorescent lyophilized beads (BD Biosciences, San Diego, CA, USA) to obtain the absolute numbers and estimate the size of the EVs. EVs isolated from doxorubicin treatment were stained with FITC-conjugated annexin V (BioLegend, San Diego, CA, USA) in 1× annexin V binding buffer (BD Pharmingen^TM^, San Diego, CA, USA) for 15 min at room temperature. After that, known numbers of BD Trucount^TM^ fluorescent lyophilized beads were added, followed by flow cytometry analysis. The numbers of annexin V^+^ EVs were calculated using the following formula:

$$Annexin\;V^{+}\mathrm{EV}=\frac{{\mathrm{Events}\;\mathrm{of}\;\mathrm{Annexin}\;V}^{+}\mathrm{EV}}{\mathrm{Events}\;\mathrm{of}\;\mathrm{beads}}\;\times \;\frac{\mathrm{Bead}\;\mathrm{counts}\;\mathrm{per}\;\mathrm{tube}}{\mathrm{Sample}\;\mathrm{volume}}$$

### 2.12. Statistical Analysis

All the data are represented as the means ± SD of three independent experiments. Statistical analysis for the significance between two groups was performed using a Student’s *t*-test in GraphPad Prism v8.0.4 (GraphPad Software Inc., San Diego, CA, USA). A *p*-value less than 0.05 was considered statistically significant.

## 3. Results

### 3.1. Doxorubicin Enhances Chemoresistance and PD-L1 Expression in Osteosarcoma Cells

**Figure 1:**
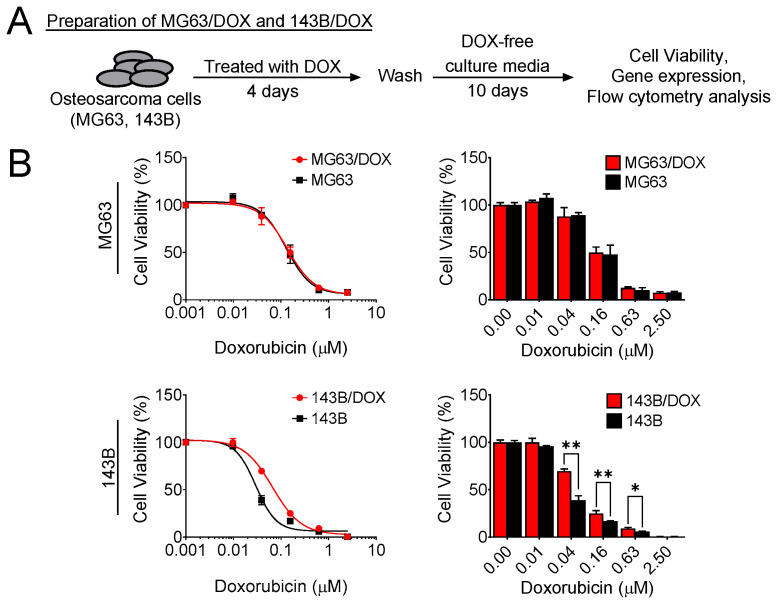
Doxorubicin enhances chemoresistance in osteosarcoma cells. (**A**) Schematic of MG63/DOX and 143B/DOX cell preparation. (**B**) MG63/DOX and 143B/DOX cell viability after treatment with doxorubicin. Cells were treated with various concentrations of doxorubicin for 4 days, and then the MTS assay was performed to analyze cell viability. The data represent the means ± SD. * *p* < 0.05 and ** *p* < 0.01 vs. their parental cells.

To determine whether chemotherapy mediated chemoresistance in osteosarcoma, we treated MG63 and 143B osteosarcoma cells with doxorubicin for 4 days, followed by further culture for an additional 10 days in fresh culture medium without doxorubicin; these cells were named MG63/DOX and 143B/DOX, respectively (Figure 1A). On day 14, we evaluated the doxorubicin sensitivity of MG63/DOX and 143B/DOX compared with their parental cells using the MTS assay. Only 143B/DOX cells showed more resistance to doxorubicin than their parental control (Figure 1B).

**Figure 2:**
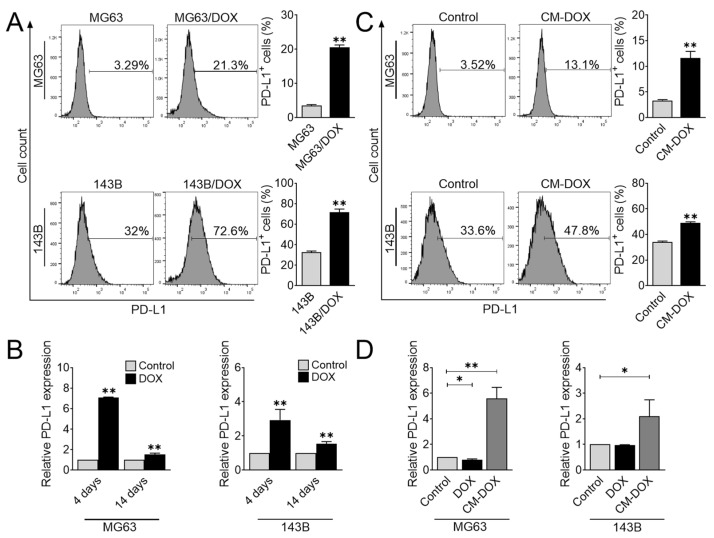
Doxorubicin accelerates PD-L1 expression in osteosarcoma cells. (**A**) Flow cytometric analysis of PD-L1^+^ osteosarcoma cells. MG63/DOX and 143B/DOX were analyzed for cell surface expression of PD-L1 using flow cytometry. The data represent the means ± SD. **, *p* < 0.01 vs. their parental cells. (**B**) PD-L1 gene expression in response to doxorubicin treatment. Osteosarcoma cells were treated with doxorubicin (40 nM) for 4 days, washed, and further cultured for an additional 10 days. The qRT-PCR was performed to evaluate PD-L1 gene expression on days 4 and 14. The data represent the means ± SD. **, *p* < 0.01 vs. control (0.04% DMSO). (**C**) Flow cytometric analysis of PD-L1^+^ osteosarcoma cells. MG63 and 143B cells were treated with CM-DOX for 48 h and then were analyzed for PD-L1 protein expression using flow cytometry. The data represent the means ± SD. **, *p* < 0.01 vs. control. (**D**) PD-L1 gene expression after CM-DOX treatment. Osteosarcoma cells were treated with CM-DOX or doxorubicin (40 nM) for 24 h, and qRT-PCR was performed to determine PD-L1 gene expression. The data represent the means ± SD. *, *p* < 0.05 and **, *p* < 0.01 vs. control (0.04% DMSO).

Given the importance of PD-L1 in tumor recurrence and immunosuppression, we studied whether PD-L1 expression may be upregulated by standard chemotherapy treatment by examining cell surface PD-L1 expression in MG63/DOX and 143B/DOX by flow cytometry. The percentage of PD-L1 positive cells was higher in both cell lines than that in their control counterparts (Figure 2A). Consistently, we also observed the upregulation of PD-L1 gene expression on days 4 and 14 after doxorubicin treatment (Figure 2B). Notably, PD-L1 expression after chemotherapy was also observed recently in clinical osteosarcoma tissue samples [26]. However, the underlying mechanisms of PD-L1 regulation following chemotherapy treatment remain elusive.

To gain insight into the mechanism of how doxorubicin induces PD-L1 expression, we studied whether doxorubicin stimulated osteosarcoma cells to secrete cytokines/growth factors that activated PD-L1. To this end, we treated osteosarcoma cells with doxorubicin for 4 days, washed them, and continued the culture in doxorubicin-free culture media for another 3 days. The drug-free medium was collected and subsequently used to treat osteosarcoma cells. The conditioned medium derived from doxorubicin treatment substantially enhanced the gene and protein expression of PD-L1 (Figure 2C,D). Although previous studies demonstrated that doxorubicin directly upregulated PD-L1 expression in osteosarcoma [26], we also considered the possibility of indirect regulation. To ensure that the upregulation of PD-L1 was not due to the effect of doxorubicin in the culture medium, we treated osteosarcoma cells with doxorubicin for 1 day and examined PD-L1 gene expression. As expected, doxorubicin itself was not effective for regulating PD-L1 expression (Figure 2D). Taken together, these data indicated that doxorubicin modulates PD-L1 expression by an indirect mechanism, possibly through stimulating cytokine/growth factor secretion.

### 3.2. Doxorubicin Treatment Stimulates Osteosarcoma Cells to Secrete Proinflammatory Cytokines

**Figure 3:**
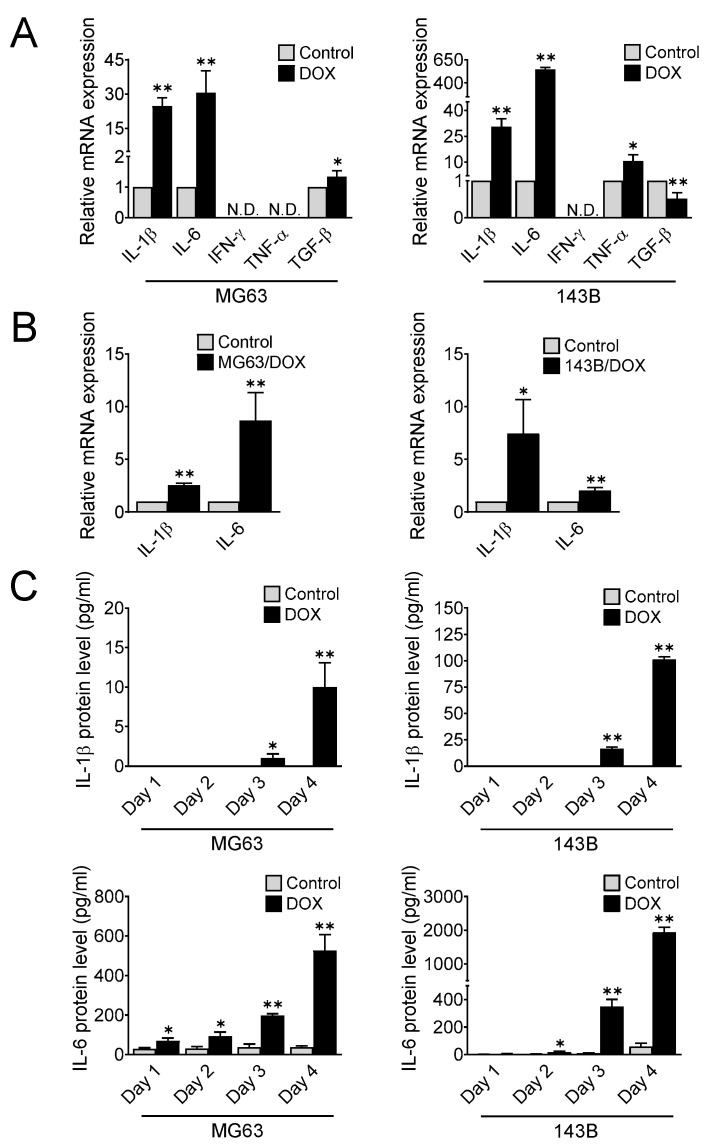
Doxorubicin enhances the expression of proinflammatory cytokines IL-1β and IL-6 in osteosarcoma. (**A**) Gene expression of the proinflammatory cytokines IL-1β, IL-6, IFN-γ, TNF-α, and TGF-β after doxorubicin treatment. Human osteosarcoma cells were treated with doxorubicin (40 nM) for 4 days, and qRT-PCR was performed to determine cytokine gene expression (N.D., not detected). The data represent the means ± SD. *, *p* < 0.05 and **, *p* < 0.01 vs. control (0.04% DMSO). (**B**) IL-1β and IL-6 gene expression in MG63 and 143B cells after doxorubicin withdrawal for 10 days. The data represent the means ± SD. *, *p* < 0.05 and **, *p* < 0.01 vs. untreated parental cells. (**C**) Secretion of IL-1β and IL-6 after doxorubicin treatment. Osteosarcoma cells were treated with doxorubicin (40 nM), and the culture media were collected on days 1, 2, 3, and 4 to determine the IL-1β and IL-6 levels using ELISA. The data represent the means ± SD. *, *p* < 0.05 and **, *p* < 0.01 vs. control (0.04% DMSO).

Inflammation is a hallmark of cancer that plays a role in tumor progression, including cell proliferation, migration, chemoresistance, and immunosuppression [29]. Proinflammatory cytokines such as TNF-α, IL-6, IL-1β, TGF-β, and IFN-γ modulate PD-L1 expression in both cancer and stromal cells [30,31]. To determine whether doxorubicin enhanced TNF-α, IL-6, IL-1β, TGF-β, and IFN-γ expression in osteosarcoma, we treated MG63 and 143B cells as indicated (Figure 1A). On days 4 and 14, the osteosarcoma cells were collected to determine the gene expression of the indicated cytokines. Treatment with doxorubicin dramatically induced the expression levels of IL-6 and IL-1β genes in both the MG63 and 143B cells (Figure 3A), and the levels remained high even after doxorubicin withdrawal on day 14 (Figure 3B). However, a controversial finding was observed in the TNF-α and TGF-β gene expression between both cell lines. Although TGF-β gene expression was significantly increased in MG63 cells after doxorubicin treatment, its level was reduced in 143B cells. Similarly, TNF-α gene expression was enhanced in 143B cells but undetectable in MG63 cells. Additionally, we could not detect the induction of the IFN-γ gene in either cell line (Figure 3A). We next examined the level of IL-1β and IL-6 in MG63 and 143B cells on days 1, 2, 3, and 4 after doxorubicin treatment. Consistent with the induction of gene expression, ELISA showed a clear induction of IL-6 and IL-1β protein in the culture medium of the doxorubicin treatment group (Figure 3C). These results suggested the massive secretion of inflammatory cytokines IL-6 and IL-1β after chemotherapy treatment.

### 3.3. IL-1β in Response to Doxorubicin Induces PD-L1 Expression

**Figure 4:**
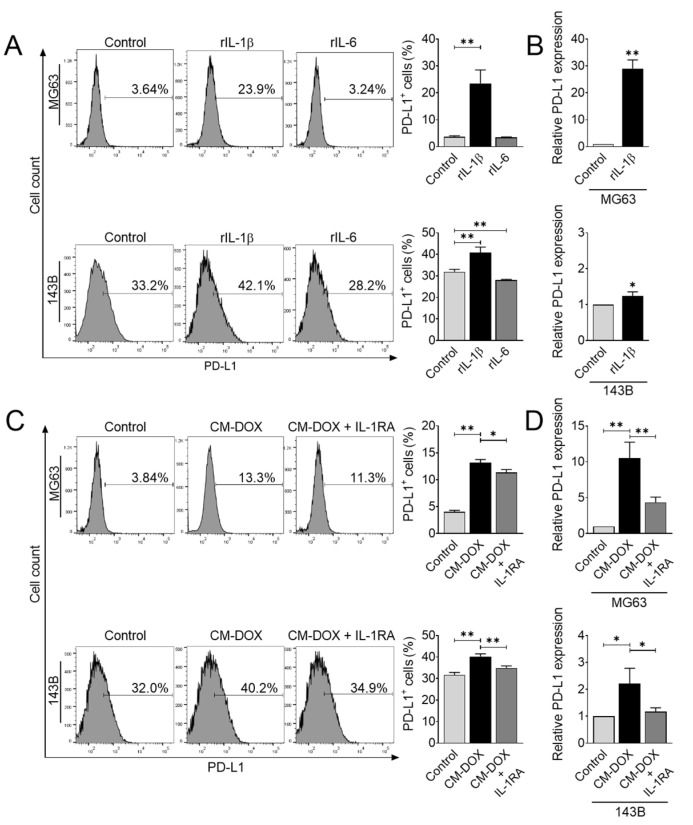
IL-1β secretion in response to doxorubicin upregulates PD-L1 expression. (**A**) Flow cytometric analysis of PD-L1^+^ cells after recombinant IL-1β or IL-6 treatment. Human osteosarcoma cells were treated with rIL-1β (10 ng/mL) or rIL-6 (20 ng/mL) for 48 h, and PD-L1 protein expression was analyzed using flow cytometry. The data represent the means ± SD. **, *p* < 0.01 vs. control. (**B**) PD-L1 gene expression after rIL-1β treatment. Osteosarcoma cells were treated with rIL-1β (10 ng/mL) for 24 h, and qRT-PCR was performed to determine PD-L1 gene expression. The data represent the means ± SD. *, *p* < 0.05 and **, *p* < 0.01 vs. control. Flow cytometric (**C**) and qRT-PCR (**D**) analyses of PD-L1 expression after CM-DOX treatment. Osteosarcoma cells were treated with CM-DOX in the presence or absence of IL-1RA (100 ng/mL) and analyzed for PD-L1 expression. The data represent the means ± SD. *, *p* < 0.05 and **, *p* < 0.01 vs. control.

To directly test whether IL-1β and IL-6 increased PD-L1 expression, we treated MG63 and 143B cells with recombinant human IL-1β and IL-6, and determined the gene and cell surface expression of PD-L1. Exposure of IL-1β was sufficient to increase PD-L1 gene and protein expression in both cell lines (Figure 4A,B). In contrast, we found a slight reduction in PD-L1 in 143B cells after treatment with IL-6 (Figure 4A). These results suggested that IL-1β is the most critical upstream signal that may modulate PD-L1 levels in osteosarcoma cells.

Exposure to recombinant human IL-1β caused a significant increase in PD-L1 expression. Next, we confirmed whether the induction of IL-1β in conditioned medium derived from doxorubicin affected the PD-L1 levels in osteosarcoma. To this end, we utilized the IL-1 receptor antagonist to competitively inhibit the binding of IL-1β to the IL-1 receptor. Osteosarcoma cells were treated with doxorubicin-derived culture medium in the absence or presence of the IL-1 receptor antagonist. We found that the IL-1 receptor antagonist significantly inhibited PD-L1 gene and protein expression induced by doxorubicin-derived culture medium (Figure 4C,D). Our data suggested that IL-1 signaling plays a central role in modulating PD-L1 expression during and after chemotherapy treatment.

### 3.4. Doxorubicin Promotes the Release of EVs

**Figure 5:**
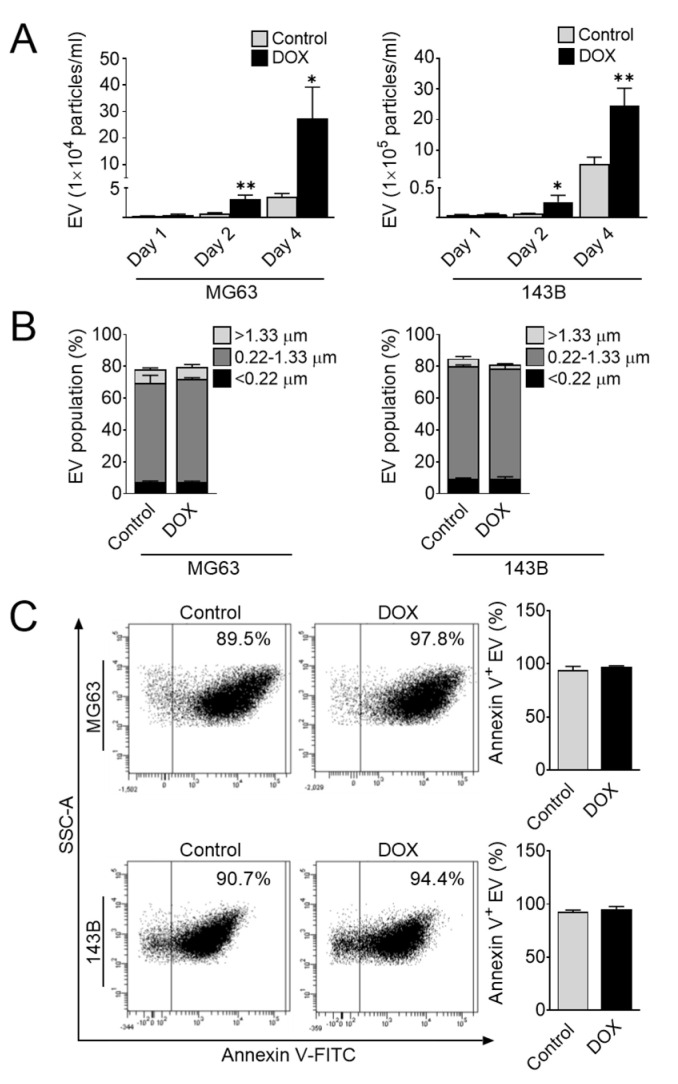
Doxorubicin promotes the release of EVs in osteosarcoma cells. Effect of doxorubicin on EV secretion from osteosarcoma cells. (**A**) Quantification of EV at days 1, 2, and 4 after doxorubicin treatment. (**B**) Representative size distribution estimation of EV before and after doxorubicin treatment. (**C**) Representative dot plot of annexin V^+^ EV by flow cytometry. The data represent the means ± SD. *, *p* < 0.05 and **, *p* < 0.01 vs. control (0.04% DMSO).

Given the extensive induction of proinflammatory cytokines IL-1β and IL-6 following doxorubicin treatment, we subsequently studied whether the generation of EVs was necessary for IL-1β and IL-6 expression. We first investigated the effect of doxorubicin on EV release in osteosarcoma cells. To this end, we treated MG63 and 143B cells with doxorubicin for 1, 2, and 4 days. At each time point, the supernatant was collected, and then the amount and size of EVs were determined by flow cytometry. We found extensive release of annexin-V-positive EVs in a time-dependent manner, and the amount of EVs following doxorubicin treatment was significantly increased compared with that in the untreated control cells (Figure 5A). Considering the size of EVs, we next estimated the size distribution of EVs in osteosarcoma cells after doxorubicin treatment. We also found that the major population of EVs secreted from these cells ranged from 0.22 to 1.33 µm, corresponding to the size of microvesicles (Figure 5B). However, no differences were observed in the size or annexin-V-positive population in both the treated and control cells (Figure 5B,C). Collectively, these observations clearly suggested that doxorubicin enhances EV release in osteosarcoma cells. Consistent with the induction of EVs, the levels of IL-1β and IL-6 were significantly increased in doxorubicin-treated culture medium (Figure 3C). These findings suggested a significant correlation between the number of EVs and the levels of proinflammatory cytokines IL-1β and IL-6.

### 3.5. Doxorubicin-Derived Extracellular Vesicles Modulate IL-1β and IL-6 Expression

**Figure 6:**
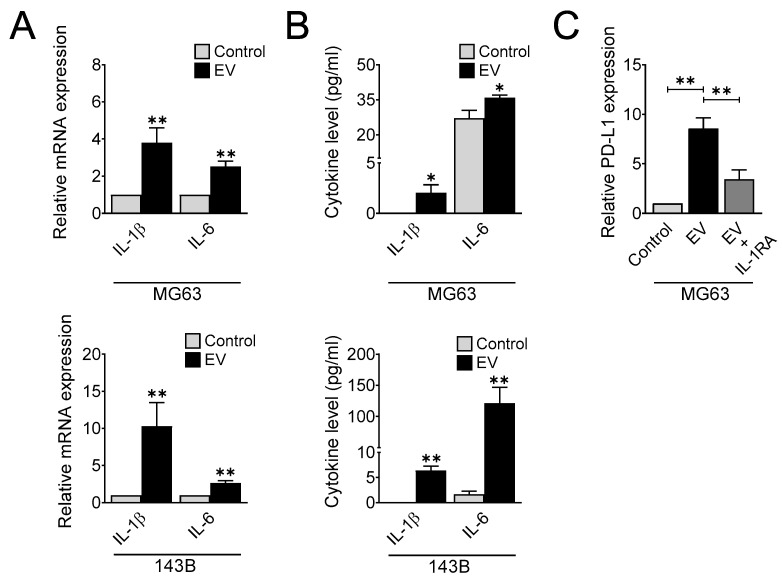
Doxorubicin-derived extracellular vesicles regulate IL-1β and IL-6 expression. IL-1β and IL-6 gene (**A**) and protein (**B**) expression after EV treatment. Human osteosarcoma cells were treated with 50 µg/mL of EVs and analyzed for IL-1β and IL-6 gene and protein expression by qRT-PCR and ELISA, respectively. The data represent the means ± SD. *, *p* < 0.05 and **, *p* < 0.01 vs. control. (**C**) PD-L1 gene expression after EV treatment. Osteosarcoma cells were treated with 100 µg/mL of EV in the presence or absence of IL-1RA (100 ng/mL) for 24 h, and qRT-PCR was performed to determine PD-L1 gene expression. The data represent the means ± SD. **, *p* < 0.01 vs. control.

To determine the importance of EVs in the setting of inflammation in osteosarcoma after chemotherapy, MG63 and 143B cells were treated with EVs and subjected to the quantification of IL-1β and IL-6 gene and protein expression by qRT-PCR and ELISA, respectively. As shown in Figure 6A, EVs substantially induced the mRNA expression of IL-1β and IL-6 compared with the control in MG63 and 143B cells. Additionally, a significant increase was observed in the protein levels of IL-1β and IL-6 in the culture medium of MG63 and 143B cells treated with EVs (Figure 6B). These findings indicated that EVs generated by doxorubicin enhance the expression of IL-1β and IL-6 in osteosarcoma.

We next evaluated whether EV treatment is sufficient for modulating PD-L1 expression. To this end, we treated MG63 cells with EVs in the presence or absence of the IL-1 receptor antagonist for 1 day and determined the levels of PD-L1 gene expression. Our results revealed that EVs strongly upregulated PD-L1 expression, and cotreatment with the IL-1 receptor antagonist abolished this effect (Figure 6C). These results strongly suggested that EVs accelerate PD-L1 expression via IL-1 signaling.

## 4. Discussion

Based on our current findings, we present a model showing that treatment after chemotherapy leads to a chemoresistance phenotype with increasing PD-L1 expression. Mechanistically, doxorubicin stimulates the release of cancer-derived EVs, which further promotes IL-1 production to subsequently activate PD-L1 expression in osteosarcoma. This pathway may represent an intrinsic adaptive mechanism of tumor recurrence.

The impact of PD-L1 on tumor immune escape is widely documented, and the inflammatory microenvironment was proposed to augment its expression [30]. Here, we showed that doxorubicin treatment upregulated PD-L1 levels, and the expression was retained even two weeks after treatment in osteosarcoma. Inflammatory signaling can also be regulated by tumor cells to increase PD-L1 expression, thereby suppressing T-cell function in the tumor microenvironment. IFN-γ is considered a potent inducer of PD-L1 [32]. However, because we observed no increase in its expression following doxorubicin treatment, we wondered whether other signaling pathways might exist in this process. Based on a previous report, IL-1β, IL-6, TNF-α, and TGF-β are also involved in upstream signaling for PD-L1 expression [30]. Our data indicated that doxorubicin enhanced IL-1β and IL-6 levels in osteosarcoma. We showed that IL-1β, but not IL-6, is involved in regulating PD-L1 expression. However, another study demonstrated that IL-6 controls PD-L1 expression through post-translational modification by inducing PD-L1 glycosylation necessary for its stability in hepatocellular carcinoma [33]. These conflicting results may have been due to the different transcriptional and translational signals used by the different cell types. Although IL-6 is insufficient to modulate PD-L1 expression, it may be implicated in different functional roles in this osteosarcoma model. Although the results presented here focused on the role of IL-1β, it is possible that other cytokines or growth factors are functionally important for modulating PD-L1 expression.

Chemotherapy regulates PD-L1 expression in several types of cancers. In osteosarcoma, the level of PD-L1 was upregulated in both clinical tissues and cell lines after doxorubicin treatment [26]. Consistent with this evidence, our study demonstrated that the expression of PD-L1 was markedly increased in 143B/DOX and MG63/DOX cells compared with that in parental cells, and 143B/DOX cells showed an increased resistance to doxorubicin treatment. Although the role of PD-L1 in regulating the immune response by modulating T-cell function is extensively highlighted, recent evidence has demonstrated a distinct role of PD-L1 in chemoresistance in which the downregulation of PD-L1 sensitized chemoresistant cells to chemotherapy [34,35]. It is likely that the combined effect of PD-L1 on immune modulation and chemoresistant functions may contribute to tumor recurrence after chemotherapy.

Accumulating evidence has shown that cytokines such as IL-6, IL-8, C-C motif chemokine ligand 2, and colony stimulating factor 2 are markedly secreted after chemotherapy treatment [18,36]. However, the role of chemotherapeutic drugs in enhancing inflammation in cancer is still unclear. Interestingly, we showed that doxorubicin significantly increased the amount of EVs released from osteosarcoma cells. These EVs served as mediators in stimulating inflammatory cytokine IL-1β and IL-6 production. EVs mediate cell-to-cell communication, and function as messengers by carrying a cargo of proteins, nucleic acids, and lipids, which play critical roles in tumor progression [4]. Chemotherapeutic agents have been reported to stimulate the secretion of EVs containing various biologically active substances, such as ABCB1, annexin A6, miR-9-5p, miR-203a-3p, and miR-195-5p, promoting chemoresistance and cancer invasiveness [6,8,37]. In addition to transferring their contents, EVs can activate cell signaling pathways by ligand/receptor interactions; for example, EVs carrying mitochondrial DNA can activate the proinflammatory signal in recipient cells [38]. Additionally, EVs harboring damage-associated molecular pattern molecules from injured and stressed cells function as a danger signal in contributing to inflammation [39]. In the present study, we showed the relationships between cancer-derived EVs and proinflammatory cytokine induction. Identification of the change in the content of the EVs in response to doxorubicin may be further used to assess how EVs regulate proinflammatory cytokine expression in osteosarcoma.

In conclusion, we have shown that EV secretion in response to doxorubicin enhances PD-L1 expression via the IL-1 signaling pathway. This finding provides the missing mechanistic link between chemotherapy and PD-L1 expression, which plays an important role in tumor recurrence. Because PD-L1 suppresses T-cell function in tumor immune escape, our results suggested that the combination of chemotherapy regimens that target PD-L1 and IL-1 signaling is an effective strategy for osteosarcoma treatment.

## Data Availability

The data supporting the findings of this study are available from the corresponding author.
